# Sharing of proximal fibers by the anterolateral and lateral collateral ligaments in the human knee: a cadaveric study

**DOI:** 10.1038/s41598-023-38211-9

**Published:** 2023-07-29

**Authors:** Ashutosh Kumar, Khursheed Raza, Hare Krishna, Ravi K. Narayan, Rakesh K. Jha, Chiman Kumari, Adil Asghar, Tarun Kumar, Prabhat Agrawal

**Affiliations:** 1 Department of Anatomy, All India Institute of Medical Sciences, Patna, Bihar 801507 India; 2 Department of Anatomy, All India Institute of Medical Sciences, Deoghar, Jharkhand India; 3 Department of Anatomy, All India Institute of Medical Sciences, Jodhpur, Rajasthan India; 4 Department of Anatomy, Dr. B.C. Roy Multi Speciality Medical Research Center, Indian Institute of Technology, Kharagpur, West Bengal India; 5 Department of Anatomy, Postgraduate Institute of Medical Education and Research, Chandigarh, India; 6 Department of Pathology, All India Institute of Medical Sciences, Patna, Bihar India; 7 Department of Orthopedics, All India Institute of Medical Sciences, Patna, Bihar India

**Keywords:** Anatomy, Medical research

## Abstract

Literature is highly inconsistent in describing the proximal attachment of the anterolateral ligament (ALL) and its relationship with the lateral collateral ligament (LCL) in human knees. This observational study aims to investigate that lacuna. The gross dissection was performed in the lower limbs (n = 83) from the donated adult-age (> 18 years) embalmed cadavers from medical institutions in the north and east India. The dissected knee specimens were first examined macroscopically. Further routine and special staining and microscopic examinations were performed. The ALL was absent in approximately 20.4% of the studied knee specimens (17/83). In remaining, the sharing of ALL and LCL proximal fibers was observed as a consistent finding (~ 97%) with rare exceptions. The mean length of the tibial and meniscal limbs of ALL was 1.57 ± 0.8 cm [Range (R) 0.5–4 cm] and 0.73 ± 0.47 cm [Range (R) 0.1–1.6 cm], respectively. In addition, multiple variations in its presentation were observed. We propose that the proximal sharing of LCL-ALL fibers is a dominant feature in the studied population. The sharing of the fibers may impact the biomechanics and injury mechanisms for both ligaments. The possibility of ethnic variations in the ALL morphology should be a concern during reconstruction surgery.

## Introduction

Recent studies consistently described the presence of an obliquely placed ligamentous structure—anterolateral ligament (ALL)—on the lateral aspect of the knee^1–3^. ALL is said to have a crucial role in maintaining anterior translational and anterolateral rotational stability at the knee joint^4^. ALL functions complement the anterior cruciate ligament (ACL), and its injury is often accompanied by the latter^5–7^. Studies have shown that sectioning of the ALL significantly increases anterior translation and internal rotation in the early phase of the pivot shift in ACL ruptured knees^8,9^. A failure to detect associated ALL injury is common for residual knee joint instability following ACL repair^5–7^.

The ambiguity over the existence of ALL persisted for more than a century^10–15^. Only in the current decade were its anatomical details brought to the fore^16–18^. The knowledge of ALL anatomy evolved from its first description by Paul Segond in 1879 as a “pearly, resistant, fibrous band”^10^ to “a component of the knee joint capsule”^11,12^ or “capsulo-osseous layer of the iliotibial tract (ITT)”^13,19–21^ to a truly ligamentous structure^17,22,16^ connecting lateral femoral epicondyle to the lateral condyle of the tibia^17^. A detailed description of ALL was given by Claes et al. in 2013^17^, who found that ALL attaches proximally on the lateral femoral epicondyle and distally on the lateral meniscus and the tibia midway between Gerdy’s tubercle and the fibular head. Of note, the recent studies are broadly uniform in describing the distal meniscal and tibial attachments. However, they reported its variable proximal attachment to the femur and its relationship with the lateral collateral ligament (LCL)^16,17,23,24^. Vincent et al.^16^ described the proximal attachment of ALL from the lateral femoral condyle instead of the lateral epicondyle.

Moreover, Irvine et al.^23^ and Campos et al.^24^ described ALL as an anterior oblique band from LCL. In a recent study, Olewnik et al*.* reported ALL starting from the LCL in 12.9% of the adult Caucasian cadavers containing this ligament^18^. Interestingly, Claes et al.^17^ also indicated the proximal sharing of the ALL fibers with LCL, nearer to their origin. However, they did not describe it as emanating from LCL.

A precise knowledge of the proximal attachment of ALL and its relationship with LCL is crucial for explaining the biomechanics and injury mechanisms and formulating surgical reconstruction and rehabilitative approaches for this ligament^3,25^. Therefore, it needs to be further studied. In this study, we aim to investigate that lacuna in the literature.

## Materials and methods

### Study type

An observational study.

### Cadaveric dissection and gross examination

Eighty-three knee joint specimens were dissected from the adult age (> 18 years) cadavers embalmed with 10% formalin. Out of the total specimens examined, there were 53 unpaired specimens from the detached limbs and thirty paired specimens from intact adult cadavers (nine male and six female, mean age = 74.1 (± 7.5) years, Range (R) = 65–90 years). The cadavers were obtained through the body donation programs of the medical institutes in north and east India under the ‘Body Donation Program’ regulated by the Anatomy Act, 1959, Government of India, and permitted for research and educational use. Specimens were grossly screened by a team of trained anatomist, pathologist, and orthopedist for signs of osteoarthritis, fractures, existing ACL injury, congenital deformity, infections, and previous surgeries.

Dissection of the knee joint was done per the protocol given in Cunningham’s dissection manual (Volume 1)^26^. First, the skin around the knee joint was incised and reflected, followed by the removal of the soft tissue around the knee joint, especially around the ITT, the short head of the biceps femoris, and the lateral aspect of the knee. Next, the dissection field was cleared of subcutaneous fat. Then, the iliotibial tract was cut from the insertion at Gerdy’s tubercle on the tibia and reflected. After that, we cleared soft tissue around the knee joint, which led to visualization of LCL extending from the lateral epicondyle of the femur to the head of the fibula. Next, the attachment of ALL with lateral meniscus was approached by incising the joint capsule.

Further, the anatomical relationship of ALL with the surrounding structures, like lateral meniscus, the lateral inferior genicular vessels, the LCL, and the popliteus tendon, were noted. Attachments of the ALL, LCL, ITT, and popliteus tendon were then delineated and marked with colored metal pins or paint. Finally, we provide a video description of the dissection procedure applied for this study (Supplementary Files 1, 2).

Anatomical characterization of ALL, in terms of distinct proximal and distal attachments, sharing of fibers with the LCL, and lateral meniscus, was performed for each specimen. The ALL was excised from the cadaveric knee specimens *en bloc*. The proximal attachments of LCL and ALL were resected from the lateral epicondyle of the femur. The LCL and ALL were then sectioned distally at their fibular, meniscal, and tibial attachments. Bone blocks or meniscal tissue present at the site of the attachments were included in the section. This specimen was used for histologic analysis.

### Morphometric evaluation

The length measurements were taken for the distal unshared/free components of ALL: tibial and meniscal limbs in the dissected specimen with the help of a sliding digital caliper with a precision set at 0.01 mm. The measurement points were placed at the bifurcation site of the ligament and distal attachments on the lateral meniscus or tibia. For standardization, care was taken to take the measurements in the extended knee with the foot in neutral rotation to prevent the ligament’s overstretching, shortening, or bending. The presence of any of the two components, either tibial or meniscal limb was considered proof for ALL. Results were expressed as mean (± SD).

### Histological and microscopic examination

Based on the observations in the gross examination, representative specimens were processed for histological and microscopic examination and evaluated by a team of anatomy and pathology experts from the investigators. The longitudinal thick tissue sections were cut from *en bloc* specimens of joined LCL and ALL and mounted over glass slides. They were first examined unstained under a stereomicroscope at 10× for arrangement and sharing of the fibers between the two ligaments. Further, the paraffin block was prepared from the areas of interest from harvested tissue, and 5-micron-thick sections were obtained and stained with routine hematoxylin and eosin (H&E) and a special stain for demonstrating collagen fibers (Masson’s trichrome). The standard protocol was used for processing bone and ligamentous tissue and routine and special staining. The histological sections obtained for analysis included longitudinal sections of the LCL-ALL complex showing complete proximal and distal components. The stained slides were examined under a bright-field light microscope (Nikon, model: Eclipse Ci-L) and were photographed with an inbuilt digital camera.

### Statistical analysis

A priori sample size was not calculated. The convenient sampling strategy was used for the selection of samples for this study. Two independent observers took all measurements twice, and the intra-observer/inter-observer variability coefficient (Cohen’s Kappa) value of ≥ 0.8 was set as a threshold for the inclusion of the data for analysis. The average value of the measurements were recorded. The prevalence of the ALL in examined specimens and differences in morphometric measurements of its components were statistically analyzed in reference to body side /laterality. Descriptive statistics (mean length, range, and standard deviation) were noted. Shapiro–Wilk test was applied to check the normality of the data. The unpaired T-test was used to determine the statistical significance of the measurements’ differences in the normalized data, and the one-tailed Mann Whitney U test in skewed data with a 95% confidence interval (CI) and P value ≤ 0.05. The statistical tests were done using GraphPad Prism software, 2022.

### Ethics statement

We received the cadavers used for this study from voluntary donations under the ‘Body Donation Program’ regulated by the Anatomy Act, 1959, Government of India, and permitted for research and educational use. A written consent from the nearest kin of the deceased was received at the receiving institutes stating the same. No further clearance from the institute ethics committee was mandated for this study as we performed no experimentation on living animals or human beings.

## Results

### Cadaveric dissection and gross examination

**Figure 1:**
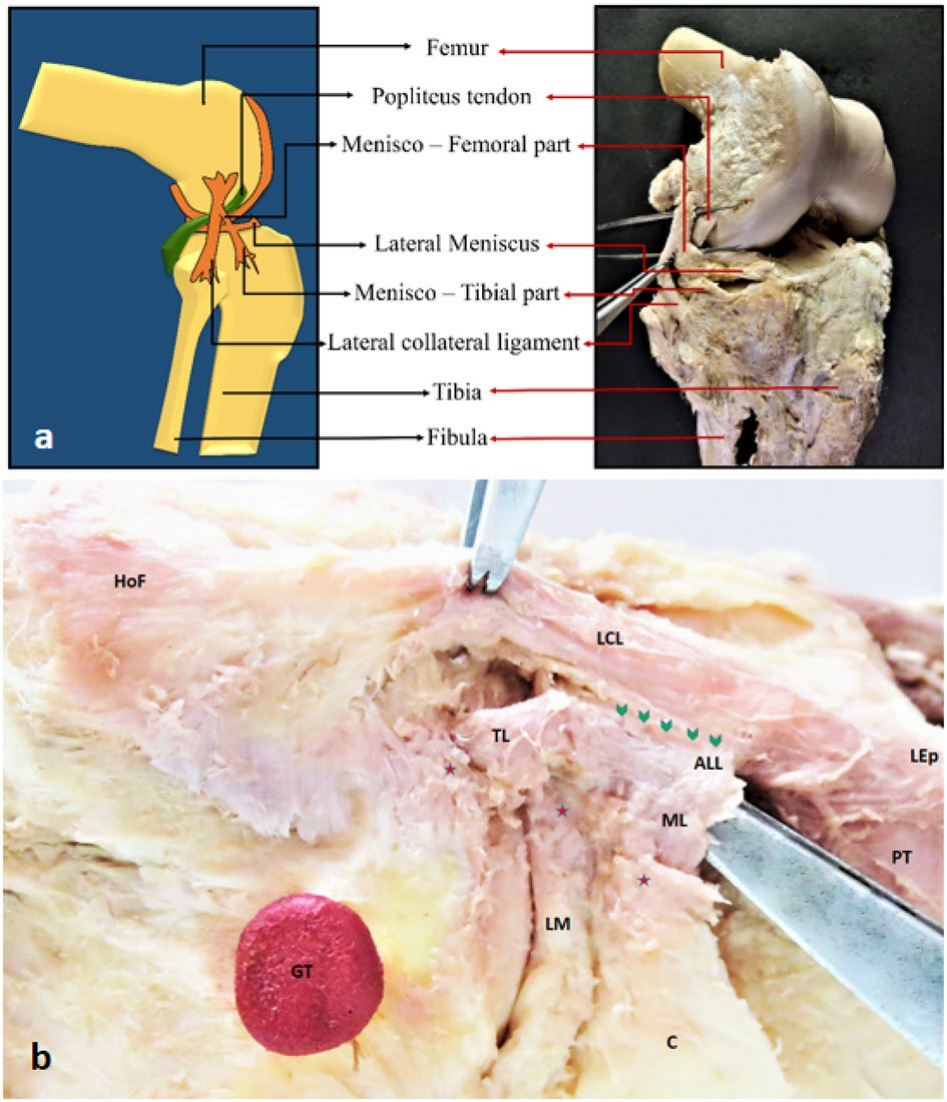
The Anterolateral ligament in a dissected specimen of the right knee. ALL appeared to originate as an oblique band from LCL due to sharing the proximal fibers in most specimens. The distal attachment of the ALL was regularly noted to be present at the midpoint between Gerdy’s tubercle and the superior tibiofibular joint closure to the upper border of the tibial condyle while giving attachment to the lateral meniscus in the way. The meniscal attachment divided the ALL into proximal—meniscofemoral and distal—meniscotibial portions (**a**). In (**b**) slight variation in the anatomy of ALL is observable as it shows extended sharing of the fibers of ALL with LCL (marked with green arrowheads). A meniscal limb is noticeable; however, it is not very distinct. The capsular attachment of the meniscal fibers was also noted. The distal attachments of the ALL (tibial and meniscal) are marked with an Asterix. *LCL* lateral collateral ligament, *ALL* anterolateral ligament, *HoF* head of the fibula, *LEp* lateral epicondyle of the femur, *GT* Gerdy’s tubercle, *TL* tibial limb, *ML* meniscal limb, *LM* lateral meniscus, *C* capsule of the knee joint, *PT* popliteus tendon.

**Figure 2:**
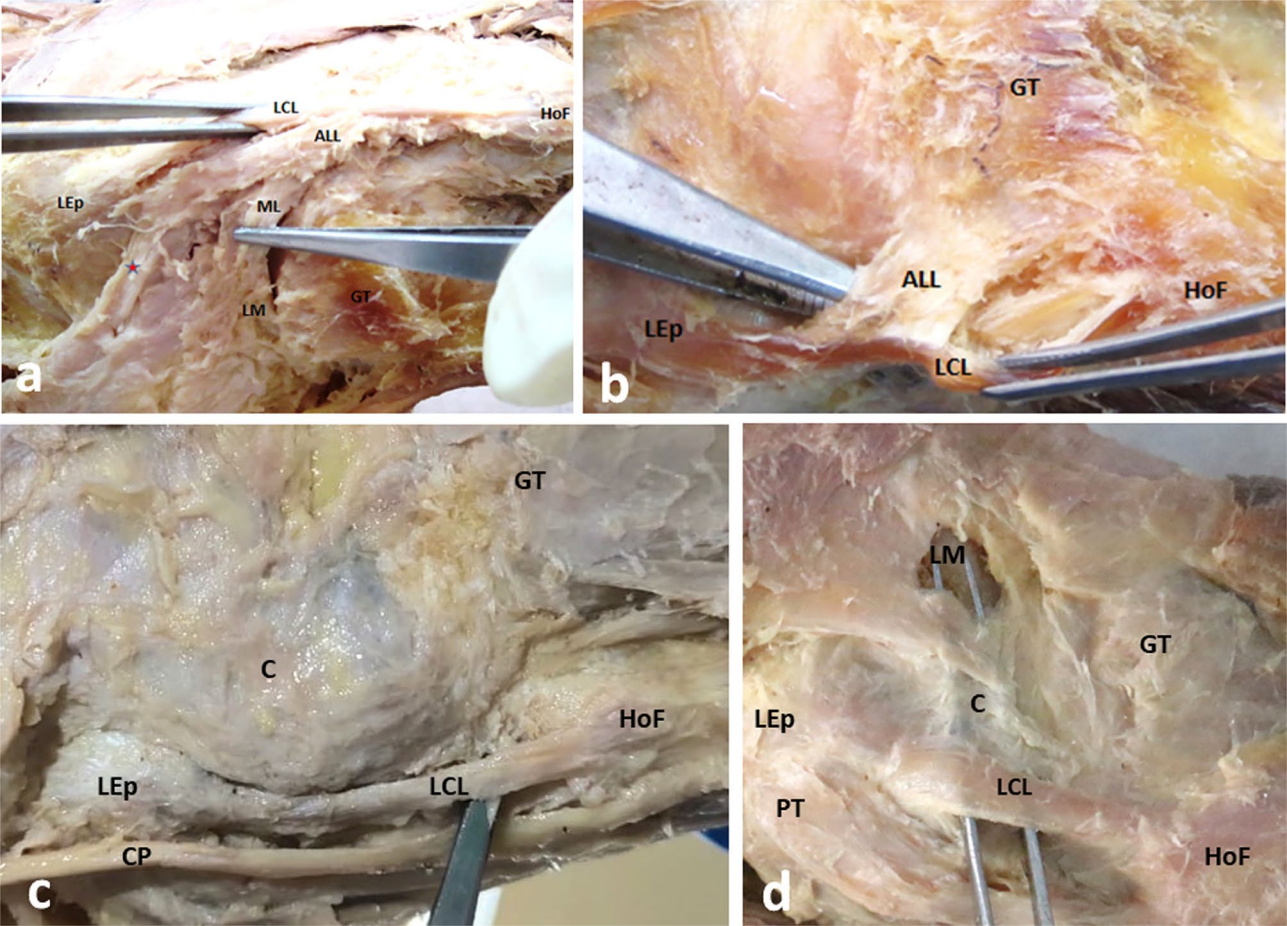
The morphological variations in presentation and cases of missing anterolateral ligament of the knee. (**a**) Inverted: The distal part of ALL can be observed, originating as an oblique band from LCL. The ligament fibers are running upward. A band of fibers is attached to the lateral meniscus-meniscal limb (ML), and the rest are attached to the knee joint capsule (marked with Asterix). (**b**) ALL can be observed as an oblique facial band from LCL and attaching to the upper tibial margin between Gerdy’s tubercle (GT) and the head of the fibula (HoF). No distal meniscal attachment of ALL was noted in this case. (**c**) A case of missing ALL in right knee. (**d**) ALL is absent. LCL is uniquely adherent to the knee joint capsule, particularly along its anterior margin, where the fibers are shared between two structures. At the posterior margin of LCL fibers were detached to facilitate entry of the forceps. No adherence with the capsule was noted on the undersurface of the ligament. *LCL* lateral collateral ligament, *ALL* anterolateral ligament, *HoF* head of the fibula, *LEp* lateral epicondyle of the femur, *GT* Gerdy’s tubercle, *TL* tibial limb, *ML* meniscal limb, *LM* lateral meniscus, *C* capsule of the knee joint, *PT* popliteus tendon, *CP* common peroneal nerve.

The ALL was present (either tibial or meniscal components) only in ~ 79.5% (66/83) of the total knee specimens. Among the paired knee specimens, the ALL was found bilaterally absent in 20% of cases (Table S1, Supplementary File 3, marked with asterix). In most of the specimens which showed the presence of ALL (~ 97%, 64/66) (Figs. 1, 2, video description: Supplementary Material 1, 2), it appeared to originate as an oblique band from LCL due to sharing of the proximal fibers, except in a few specimens (~ 3%, 2/66) where the proximal attachment of the ALL could be traced up to the lateral epicondyle with only partial sharing of proximal fibers with LCL. A cleavage plane could be macroscopically observed between the ALL and LCL specimens. However, their proximal attachments at the lateral epicondyle of the femur overlapped extensively (Fig. S1, Supplementary File 4). Additionally, it was found that the distal attachment of the ALL gave adhesion to the lateral meniscus and was located halfway between Gerdy’s tubercle and the superior tibiofibular joint closure to the upper edge of the tibial condyle (Fig. 1a). A distinct meniscal limb was evident in the ~ 10.8% (9/83) of the specimens (Fig. 1b). Other specimens lacked a meniscal connection that separated ALL into proximal—meniscofemoral and distal—meniscotibial segments (Fig. 1a, video description: Supplementary Files 1, 2). The capsular attachment of the meniscal limb fibers was also noted (Fig. 1b). The meniscal limb was found to be the only distal attachment in 2.4% of limbs (2/83).

We observed the morphological variations in the distal attachments of ALL. In a single knee (1/83), the ALL fibers were coming out from the LCL’s distal end, then ran upward, attaching to the joint capsule; and through that to the lateral meniscus. We could also appreciate the presence of a meniscal limb (Fig. 2a). Furthermore, in 2.4% of specimens (2/83), the tibial limb appeared like a flat fascial band originating from LCL (Fig. 2b).

In the specimens where ALL was found missing (Fig. 2c), in ~ 10.8% of cases (9/83) LCL was found fused with the knee joint capsule along the margins with evidence of fibers sharing (Fig. 2d).

### Morphometric evaluation

The mean length of the tibial and meniscal limbs was 1.57 ± 0.8 cm [Range (R) 0.5–4 cm] and 0.73 ± 0.47 cm [Range (R) 0.1–1.6 cm], respectively (Table S1, Supplementary File 4). Although vast differences were present between the paired specimens from the same individuals (Tibial limb, R: 0.5–4 cm, meniscal limb, R: 0.1–1.6 cm) (Table S1, Supplementary File 3, marked with asterix). The mean length of ALL (tibial component) in male and female were 1.74 ± 0.5 cm [Range (R) 0.7–2.2 cm] and 1.77 ± 0.71 cm [Range 0.6–2.8 cm], respectively. The mean thickness and width of the tibial limbs were 1.5 ± 0.98 mm [Range (R) 0.6–3.7 mm] and 6.75 ± 1.7 mm [Range (R) 4–10 mm]. The mean thickness and width of the meniscal limbs were 0.77 ± 0.9 mm [Range (R) 0.13–1.4 mm] and 2 ± 0.28 mm [Range (R) 1.8–2.3 mm].

No statistically significant inter-individual differences were noted in the length of ALL (tibial component) in terms of body side/laterality (Mann Whitney U test, P = 0.2) determined in the studied knee specimens (n = 83) and biological sex in the paired knee specimens (n = 30, Male = 18, Female = 12) from (P = 0.85) (Table S1, Supplementary File 3, marked with asterix). The meniscal components were present in insufficient number of specimens to calculate a statistically significant difference (Table S1, Supplementary File 3).

### Histological and microscopic examination

**Figure 3:**
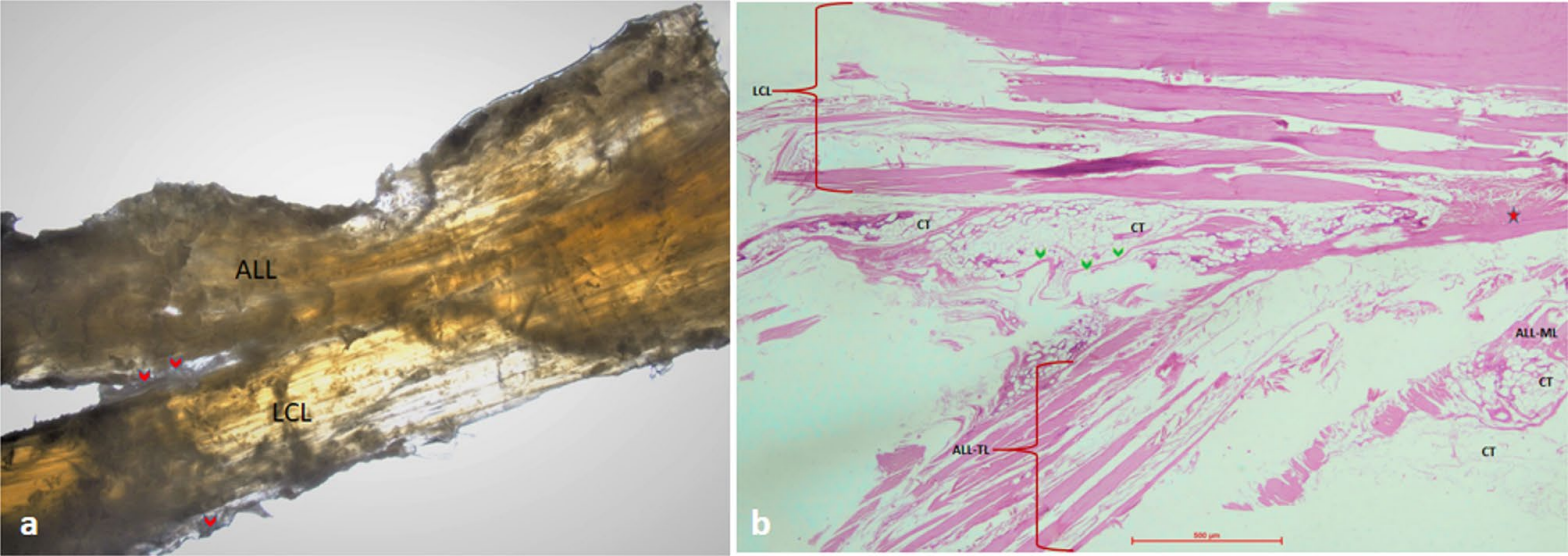
The proximal sharing observed between anterolateral ligament (ALL) and lateral collateral ligament (LCL) of the knee in histological sections. (**a**) Without staining: The proximal fibers are shared between the ligaments; however, they diverge distally. The red arrowheads show the presence of a synovial sheath around the ligaments, separating the distal parts of the ligaments. (**b**) Hematoxylin and Eosin (H&E) staining: The collagen fiber bundles from ALL are meeting proximally to that of LCL (marked with Asterix); however, they are separated distally by the presence of synovium lined (marked by green arrowheads) connective tissue (CT). *LCL* lateral collateral ligament, *ALL* anterolateral ligament, *TL* tibial limb, *ML* meniscal limb, *CT* connective tissue.

Clear evidence of sharing of the proximal fibers of ALL with LCL with no intervening synovial tissue in between was noted (Fig. 3a,b). In addition, the distal attachments of the ALL at the tibia and meniscus could be appreciated (Fig. 3b).

**Figure 4:**
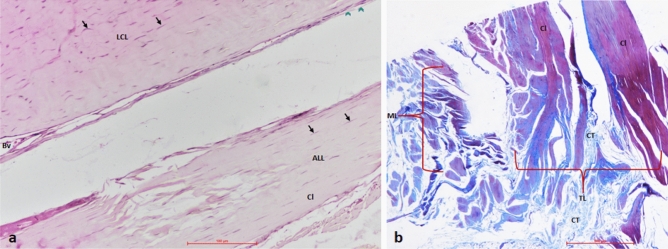
Histological evidence for ligamentous nature of anterolateral ligament (ALL). (**a**) Hematoxylin and Eosin (H&E) stained section compares distal unshared portions of the knee’s ALL and lateral collateral ligament (LCL). Similar to LCL, the parallelly arranged collagen bundles with fibroblasts dispersed in between, a distinctive feature of a ligament, can be observed. (**b**) Masson’s trichrome staining of the distal portion of ALL. The parallelly arranged collagen fiber bundles (red) forming tibial and meniscal limbs are observable. The connective tissue (CT) is stained blue. *LCL* lateral collateral ligament, *ALL* anterolateral ligament, *TL* tibial limb, *ML* meniscal limb, *Cl* collagen, *Bv* blood vessel.

The histological staining with the H&E and Masson’s trichrome confirmed the true ligamentous nature of ALL that matched with LCL (Fig. 4a,b). Parallel bands of the collagen bundles were observable, with fibroblasts dispersed in between (Fig. 4a).

## Discussion

The findings of this study are consistent with the existing studies in describing ALL’s distal meniscal and tibial attachments. However, we noted a striking difference in the presentation of the proximal attachment of ALL. We observed that the ALL and LCL in the knee shared proximal attachments on the lateral epicondyle of the femur. This observation contrasts the independent existence of ALL described in multiple earlier studies^17,22,27^. Furthermore, the proximal sharing of the fibers between ALL and LCL is not limited to their site of origin but descends downwards towards their site of insertion (the extent of sharing varied extensively between the individuals). The histological and microscopic examinations further confirmed our observations in gross cadaveric specimens. In contrast, our results make a case for a composite ligamentous structure—an LCL- ALL complex with shared proximal and divergent distal segments—resembling an inverted ‘Y’ letter. Considering this composite structure as a dominant anatomical presentation for LCL and ALL may bring a shift in the understood biomechanical functions, injury mechanisms, and reconstruction strategies for both of these ligaments.

Although the LCL-ALL complex has been scarcely reported, its precedence exists in the literature. The descriptions provided by the earlier studies by Irvine et al.^23^ and Campos et al.^24^, who described ALL as an anterior oblique band of LCL, are very close to our findings. Although interestingly, Claes et al*.*^17^ also indicated the proximal sharing of the fibers of ALL and LCL. The authors recognized that it was limited to the femoral epicondyle, where it originated, and they identified ALL as a separate ligament from ALL. Runer et al. noted their shared proximal origin in as high as 45% of dissected limbs^28^. More recently, Olewnik et al., 2018 reported the LCL-ALL complex (ALL starting from the LCL) in adult Caucasian cadavers. However, in contrast to our study, they observed this presentation in only 12.9% of the ALL specimens (n = 70)^18^. The notable high difference in the prevalence across the populations indicates the possibility of ethnicity-based differences in ALL morphology.

Apart from the proximal sharing with LCL, we also noted multiple morphological and metric variations in the distal attachments of ALL. The extent of the proximal sharing varied considerably between the individuals, which is reflected in the wide range of measurement values for the distal limbs [tibial limb: 1.57 ± 0.8 cm, Range (R) 0.5–4 cm, meniscal limb: 0.73 ± 0.47 cm, Range (R) 0.1–1.6 cm]. The inter-individual variance in the length of the distal limbs may result in the variation in the resistance to internal tibial rotation^4,29^; hence has applied importance to the ALL injury and reconstruction mechanisms^4^. It may also explain why the existing studies present conflicting results on ALL biomechanics and the benefits of reconstruction surgeries in limbs of injury^25^.

Although the meniscal attachment was a consistent finding, we observed a discrete meniscal limb in only ~ 13.6% of the specimens with ALL (9/66) (Fig. 1b). In ~ 6% (4/66) cases, only a meniscal attachment was present distally. In a single knee, ALL fibers were coming out from the distal end of the LCL. The fibers ran upward and attached to the joint capsule to the lateral meniscus (Fig. 2a). Occasionally, the tibial limb appeared like a flat fascial band expanding from LCL (2/66) (Fig. 2b). Cho and Kwak^29^, previously reported in the Korean population, the tibial limb as a flat fascial band in approximately 88% of the examined specimens. Membranous ALL were also observed in a study by Shetty et al. in south Indian population^30^. However, we found no descriptions for other variations in the existing literature. These variations in ALL anatomy will likely influence this ligament’s biomechanics, injury mechanisms, and reconstruction and rehabilitation strategies. Our results further suggest that the absence of the ALL may be a more frequent feature in the Indian population than that indicated in most of the earlier studies across the world^1,2^, as we found it absent in approximately 20.4% of the examined knee specimens.

Contrary to our results, the systematic reviews and meta-analysis studies from global data reported a high prevalence of ALL (88–96%)^1,2^. However, Cho and Kwak^29^, noted even a lower prevalence of ALL in the Korean population; they found it present in only 42.5% of the samples. Interestingly, a cadaveric study by Shetty et al. in south Indian population found it present in only 9% of the dissected knees (n = 42)^30^, which is much lower than we observed in the combined north and east Indian population (79.6%), indicating a wide inter-regional variation in the prevalence of ALL within the Indian population. However, there is a chance that Shetty et al. might have missed the cases with ALL fibers emanating from LCL, which resulted in a low prevalence of this ligament in their study. Notably, their described ALL morphological features match the conventional description for this ligament with direct proximal attachment to lateral femoral epicondyle^30^.

Notably, the length of ALL (tibial component) in our study (1.57 ± 0.8 cm [Range (R) 0.5–4 cm] is much lower than that reported in the literature (R 3.4–5.9 cm)^1^. It can be explained by the fact that in our study, in most specimens (~ 97%), the ALL stems from LCL rather than having an independent origin from lateral femoral epicondyle, the dominant form of ALL reported in the literature^17,22,27^.

We found no statistically significant inter-individual differences in the length of ALL in terms of body side/laterality (P = 0.24) and biological sex (P = 0.85). The cadaveric studies are scarce which described bodyside/laterality and sex-based difference in the global population. Of note, our results for sex-based differences contrast the available reports that described a significantly shorter ALL in females^31,32^.

The morphological variants of ALL observed in our study (Fig. 2a,b) may influence the anterolateral stability of the knee. The inverted ALL (Fig. 2a) seems a rare variant, as it has not been previously reported in the literature. Knowledge of such variants may be clinically significant in knee reconstruction surgeries, where ALL injury is a suspect.

Interestingly, we observed fusion of LCL fibers with knee joint capsule, in some specimens (~ 10.8%, 9/83) (Fig. 2d) where ALL was found missing (~ 20.4%, 17/83). We found no previous mention of this variation in LCL anatomy in the literature. Notably, per classic texts, LCL has no capsular attachment^33^. In these cases, the fusion of LCL with the capsule may create a functional replacement for missing ALL, thus having a biomechanical relevance in the anterolateral stability of the knee.

### Limitations and future directions

We did not perform any quantitative analysis of the proportion of proximal fibers shared between the ALL and LCL, which could inform more on the functional significance of this finding. Also, our study provides only limited data on the sex-based differences in the morphometric measurements of ALL; there is a possibility that it may influence the quality of results.

The morphometric measurements are likely to be confounded with the key anthropometric attributes of the individuals in a given population, such as height, girth, and body weight. Moreover, the post-mortem changes and tissue shrinkage during chemical preservation of the cadavers may alter the actual values of these morphological parameters. We suggest that the radiological imaging of living subjects may be more suitable for studying morphometric differences in ALL.

We screened the specimens for any age induced or pathological degeneration. However, this couldn’t be exclusively ruled out. Further, our study of ALL anatomy was limited to the north and east regions of Indian population; hence may not provide a global overview of its variations. However, owing to multiple contrasting studies in the available literature, it is reasonable to consider the possibility of ethnic variations in the presentation of the ALL in the global population.

## Conclusion

Based on the findings of this study, we propose that proximal sharing of LCL-ALL fibers is a dominant presentation in knee anatomy in the North and East Indian populations. The sharing of the fibers may impact the biomechanics, injury mechanisms, and surgical reconstructions of ALL and LCL. Moreover, the presence of metric and morphological variations warns against considering the uniform anatomy of ALL in all individuals. Therefore, ethnic variations in the anatomical presentation should be a concern during ALL reconstruction surgery.

## Supplementary Information

Supplementary Video 1.

Supplementary Information.

Supplementary Table S1.

Supplementary Figure S1.

## Data Availability

Datasets used/and or analyzed in the current study available from the corresponding author upon reasonable request.
