# Supplementary material for: Sharing of proximal fibers by the anterolateral and lateral collateral ligaments in the human knee: a cadaveric study
Source: Sci Rep. 2023 Jul 29;13:12317. doi: 10.1038/s41598-023-38211-9 (PMC10387103; doi:10.1038/s41598-023-38211-9)
Supplement: Supplementary file 2 — Supplementary Information. [file 41598_2023_38211_MOESM2_ESM.docx]

**Transcript of the video demonstration**

In this video we are going to demonstrate the anterolateral ligament of the knee in a dissected right lower limb and a prepared specimen.

Dissection of the knee joint was done as per protocol given in the Cunningham's dissection manual (Volume 1). Reflection of the skin was followed by removal of the soft tissue around the knee joint, especially, around the iliotibial tract, short head of biceps femoris, and lateral aspect of the knee. Then, the iliotibial tract was cut from its insertion at the Gerdy’s tubercle on the Tibia and reflected. Thereafter, we have cleared the soft tissue around the knee joint that led to the visualization of fibular collateral ligament (FCL) also called “lateral collateral ligament (LCL)” extending from lateral epicondyle of the femur to the head of the fibula. To visualize the anterolateral ligament (ALL) we will move on to the prepared specimen.

In this prepared specimen, here we can visualise the fibular collateral ligament extending from the lateral epicondyle of the femur to the head of the fibula. Here, we can visualize the cut tag of the biceps femoris. On the proximal end of the attachment of fibular collateral ligament, above we can visualize the cut tag of the lateral head of the gastrocnemius, and below this proximal attachment of the fibular collateral ligament we can visualize the tendon of Popliteus muscle attachment. The anterolateral ligament can be seen extending from the proximal end of the fibular collateral ligament to the Tibia, between the Gerdy’s tubercle and the head of the fibula. We have incised the capsule of the knee joint in order to visualize the attachment of the anterolateral ligament to the lateral meniscus of the knee. This lateral meniscus of the knee divides the anterolateral ligament into two parts, above, the menisco-femoral part, and below, the menisco-tibial part. In our dissections we have observed that the anterolateral ligament and fibular collateral ligament are the two limbs of a Y - shaped structure with a proximal common proximal stem.
