# Supplementary material for: Sharing of proximal fibers by the anterolateral and lateral collateral ligaments in the human knee: a cadaveric study
Source: Sci Rep. 2023 Jul 29;13:12317. doi: 10.1038/s41598-023-38211-9 (PMC10387103; doi:10.1038/s41598-023-38211-9)
Supplement: Supplementary file 3 — Supplementary Table S1. [file 41598_2023_38211_MOESM3_ESM.docx]

**Supplementary file 3**

**TABLE S1: The morphometry of tibial and meniscal limbs of anterolateral ligament.**

| **S. No.** | **Laterality**  (L-Left, R-Right) | **Sex**  (M-Male, F-Female) | **Age**  (in years) | **Length (in cm)** | |
| --- | --- | --- | --- | --- | --- |
|  |  |  |  | **Tibial** | **Meniscal** |
|  | L* | F | 70 | 1.5 | 0 |
|  | R* | F | 70 | 1.8 | 0 |
|  | L* | F | 70 | 0.8 | 0 |
| 4. | R* | F | 70 | 0.6 | 0 |
| 5. | L* | F | 84 | 2 | 0 |
| 6. | R* | F | 84 | 1.9 | 0 |
| 7. | L* | F | 90 | 2.5 | 0 |
| 8. | R* | F | 90 | 0 | 0 |
| 9. | L* | F | 70 | 0 | 0 |
| 10. | R* | F | 70 | 0 | 1.1 |
| 11. | L* | F | 72 | 2.8 | 0 |
| 12. | R* | F | 72 | 2 | 0 |
| 13. | L* | M | 90 | 0 | 0 |
| 14. | R* | M | 90 | 0 | 0 |
| 15. | L* | M | 70 | 0 | 0 |
| 16. | R* | M | 70 | 0 | 0 |
| 17. | L* | M | 65 | 0 | 0 |
| 18. | R* | M | 65 | 0 | 0 |
| 19. | L* | M | 70 | 0 | 0 |
| 20. | R* | M | 70 | 0 | 1.4 |
| 21. | L* | M | 74 | 2 | 0 |
| 22. | R* | M | 74 | 2.1 | 0 |
| 23. | L* | M | 70 | 1.3 | 0 |
| 24. | R* | M | 70 | 1.4 | 0 |
| 25. | L* | M | 75 | 2 | 0 |
| 26. | R* | M | 75 | 2.2 | 0 |
| 27. | L* | M | 72 | 2 | 0 |
| 28. | R* | M | 72 | 2 | 0 |
| 29. | L* | M | 70 | 0.7 | 0 |
| 30. | R* | M | 70 | 0 | 0 |
| 31. | L | - | - | 1 | 0 |
| 32. | R | - | - | 1 | 0 |
| 33. | L | - | - | 1 | 0 |
| 34. | R | - | - | 0.5 | 0 |
| 35. | R | - | - | 0.5 | 0 |
| 36. | L | - | - | 0 | 0 |
| 37. | R | - | - | 2.3 | 0 |
| 38. | L | - | - | 0.8 | 0 |
| 39. | L | - | - | 3.5 | 0 |
| 40. | L | - | - | 0 | 0.5 |
| 41. | R | - | - | 0 | 0.5 |
| 42. | L | - | - | 2.5 | 0 |
| 43. | R | - | - | 0.5 | 0 |
| 44. | R | - | - | 1.7 | 0.6 |
| 45. | R | - | - | 0 | 0 |
| 46. | R | - | - | 0.5 | 0 |
| 47. | L | - | - | 1.5 | 0 |
| 48. | R | - | - | 1.5 | 0 |
| 49. | L | - | - | 2.5 | 0 |
| 50. | R | - | - | 2.5 | 0 |
| 51. | L | - | - | 1.5 | 0 |
| 52. | R | - | - | 1.5 | 0 |
| 53. | L | - | - | 1 | 0 |
| 54. | R | - | - | 1 | 0 |
| 55. | L | - | - | 1.5 | 0 |
| 56. | R | - | - | 1.5 | 0 |
| 57. | L | - | - | 1.5 | 0 |
| 58. | R | - | - | 1.5 | 0 |
| 59. | L | - | - | 1.5 | 0 |
| 60. | R | - | - | 1.5 | 0 |
| 61. | L | - | - | 1.4 | 0 |
| 62. | R | - | - | 1 | 0 |
| 63. | R | - | - | 0 | 0 |
| 64. | L | - | - | 0 | 0 |
| 65. | R | - | - | 0 | 0 |
| 66. | L | - | - | 1 | 0 |
| 67. | R | - | - | 1 | 0 |
| 68. | L | - | - | 4 | 0.5 |
| 69. | R | - | - | 0 | 0 |
| 70. | L | - | - | 0.6 | 0 |
| 71. | R | - | - | 0.8 | 0 |
| 72. | R | - | - | 1.4 | 0 |
| 73. | R | - | - | 0.6 | 0 |
| 74. | L | - | - | 1.4 | 0 |
| 75. | R | - | - | 1.2 | 0 |
| 76. | R | - | - | 1.5 | 0 |
| 77. | L | - | - | 1.7 | 0 |
| 78. | R | - | - | 4 | 1.6 |
| 79. | L | - | - | 0 | 0 |
| 80. | L | - | - | 0.5 | 0.5 |
| 81. | R | - | - | 0.5 | 0.5 |
| 82. | L | - | - | 4 | 0 |
| 83. | L | - | - | 1.2 | 0 |

*Paired samples (taken from same individuals).
