# Supplementary material for: Sharing of proximal fibers by the anterolateral and lateral collateral ligaments in the human knee: a cadaveric study
Source: Sci Rep. 2023 Jul 29;13:12317. doi: 10.1038/s41598-023-38211-9 (PMC10387103; doi:10.1038/s41598-023-38211-9)
Supplement: Supplementary file 4 — Supplementary Figure S1. [file 41598_2023_38211_MOESM4_ESM.docx]

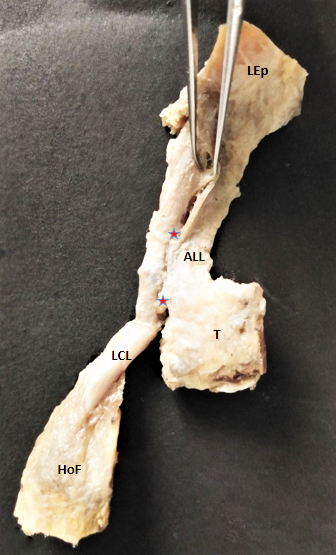


**FIGURE S 1 An *en bloc* gross specimen of LCL and ALL showing (from the anterior aspect) partial sharing of the fibers between two ligaments.** The ALL can appreciate the whole length from the lateral epicondyle (LEp) of the femur to the tibia. The proximal attachment of ALL on LEp is overlapping and indistinguishable from that of LCL. A plane of cleavage and the partial sharing of the fibers between the ligaments along the length can be macroscopically observed (marked with Asterix and a forceps). LCL-lateral collateral ligament, ALL-anterolateral ligament, HoF-head of the fibula, LEp-Lateral epicondyle of the femur, T-Tibia.
